# Elucidating the Mechanisms of Pulsed Radiofrequency for Pain Treatment

**DOI:** 10.7759/cureus.44922

**Published:** 2023-09-08

**Authors:** Jonathan De la cruz, Daniel Benzecry Almeida, Mayara Silva Marques, Ricardo Ramina, Rodolfo Jorge Fortes Kubiak

**Affiliations:** 1 Neurological Surgery, University of Antioquia, Medellín, COL; 2 Neurological Surgery, Neurological Institute of Curitiba, Curitiba, BRA; 3 Neurology, Neurological Institute of Curitiba, Curitiba, BRA; 4 Oral and Maxillofacial Surgery, Hospital Otorrinos Curitiba, Curitiba, BRA

**Keywords:** multi-modality pain management, pulsed radiofrequency treatment, pain management, pain, dorsal root ganglia

## Abstract

Pulsed radiofrequency is a well-documented treatment option for multiple painful conditions where pulses of energy are delivered close to neural elements. Since its earliest adoption, this technique has gained increasing acceptance as a minimally invasive procedure, and new applications are evolving. Studies have shown microscopic and biochemical changes that reflect beneficial effects; however, the exact mechanism of action is not yet completely understood. To redress this paucity, 11,476 articles of scientific relevance published between 1980 and November 2022 were mined through a search of the PubMed database, arriving at 49 studies both in animals and humans. In general, the experimental studies examined have shown that pulsed radiofrequency induces multiple changes with antinociceptive and neuromodulatory effects. These modifications include changes in neural and glial cells, synaptic transmission, and perineural space. Studies also reveal that pulsed radiofrequency regulates inflammatory responses, cellular signaling proteins, and the expression of genes related to pain transmission, acting in biological processes in structures such as myelin, mitochondria, axons, glial cells, connective tissue, regulation of proteins, ion channels, and neurotransmitters.

## Introduction and background

Radiofrequency (RF) is a therapeutic method using electromagnetic waves with a specific frequency. In pain management, there are two types of RFs typically aimed at neural structures: conventional RF and pulsed RF (PRF). Conventional RF was first introduced as a pain treatment in 1974 by Shealy, using an electrode and an RF generator, indicated at that time as a method to promote ablation of the dorsal root ganglion [1,2]. In this method, still in use today, ablation is induced by thermal coagulation of Aα and C fibers, occurring when temperatures above 62º Celsius are reached [3]. The constant delivery of energy promotes molecular friction in the tissue surrounding the needle, which generates heat with further thermocoagulation [1-3] Conventional RF has thus been widely used when neural destruction is desired in painful conditions, such as in trigeminal neuralgia, as well as in facet and sacroiliac pain.

In more recent decades, PRF, proposed by Sluijter in 1997 [4], became adopted as an alternative method in the treatment of several painful disorders, such as radicular pain, sacroiliac pain, discogenic pain, mechanical lumbar pain, facet pain syndrome, occipital neuralgia, cervicogenic headache, intercostal neuralgia, and whiplash injury [5-9]. In contrast to RF, in PRF the delivery of energy is not constant [10,11], usually involving electromagnetic wave pulses of 20 ms at 500 KHz close to the dorsal root ganglion or sensitive nerves, with starting durations of two to eight minutes. In these settings, temperatures usually do not rise above 42º C [11].

Initial studies involving PRF in the dorsal horn of rats did not show any major histological changes, and therefore its efficacy has been questioned [4]. Nevertheless, later studies depicted changes at the microscopic level, occurring in axonal microfilaments and microtubules of sciatic nerves of Wistar albino rats, as well as other neuronal changes preferentially at C fibers, with less effect on Aα and Aδ [12,13]. Some of these effects were shown to be potentially reversible in animal models [12]. Similarly, no morphological changes were found in early research in humans with neuropathic pain [14]. However, higher duration and voltage in PRF, when compared to conventional RF, were later shown to generate biological changes in animals and in humans [15,16]. 

A neuromodulator effect has been proposed, based mainly on different effects in animal models [13,17-19]. At the same time, the publication of positive clinical data mainly in patients with neuropathic pain has allowed PRF to be considered as a widespread technique for pain practitioners.

Despite the growing prevalence of literature that would seem to support the use of PRF in the treatment of painful disorders, there is still an important knowledge gap and much to be understood regarding its underlying mechanisms, which could help understand the complex components of pain transmission and be useful in revealing new treatment modalities. Therefore, the authors performed a systematic review of human or animal studies showing potential mechanisms of action of PRF in painful disorders.

## Review

Methods

Data Source

A judicious selection of data was based on articles and books found either in an electronic database (PubMed) or by way of the research cited in these works using Boolean Operators. The following keywords were used: radiofrequency (all fields) AND pulsed (all fields) AND mechanisms (all fields). A total of 49 articles were selected and further divided according to the primary goal and results.

Eligibility Criteria

**Figure 1:**
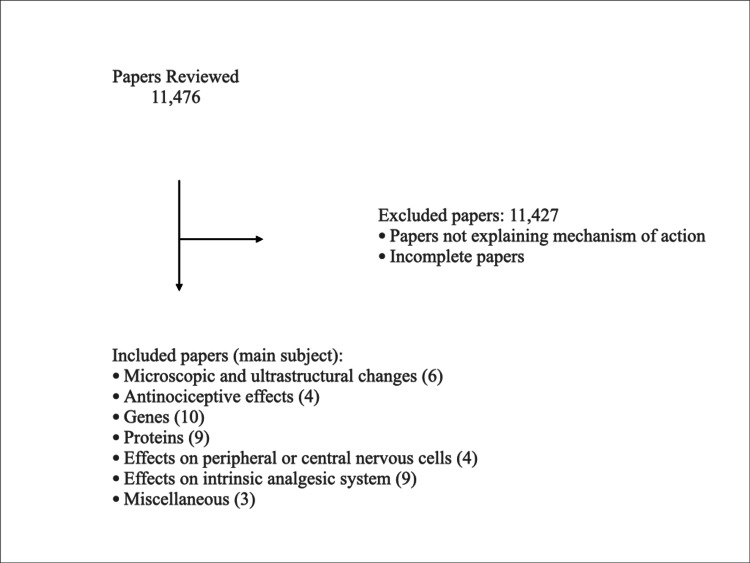
Literature review chart.

Articles were chosen based on the following inclusion criteria: (a) the article contains information about biological and nonbiological effects of RF including either animal or human investigations; (b) it is a book chapter or original papers, reviews, and case reports in scientific journals; (c) the article was published from 1980 to December 2022; (c) the article was written in English language; and (d) full-text form of the article was available (conference discussions and abstracts alone were thus excluded). These criteria are reflected in Figure 1.

The literature search rendered 11,476 articles related to PRF. After a rigorous analysis, papers that did not explain any mechanism of action of PRF as well as any incomplete papers were excluded. The final count included 49 articles, and these were then further categorized according to the results found in that specific paper. One single previous review on the mechanism of action of PRF was also included [14].

Results

General Description

The articles reviewed showed multiple mechanisms of action, encompassing the following as main topics: ultrastructural changes observed under the microscope (n=6); antinociceptive effects on animal models of pain (n=4); gene alterations (n=10); membrane protein changes (n=9); effects on peripheral and central nervous cells (n=4); alterations in endogenous opioids and other effects (n=9); reviews of the RF literature (n=4); miscellaneous (n=3). In many papers, multiple mechanisms of action could be found.

Microscopic Changes

**Figure 2:**
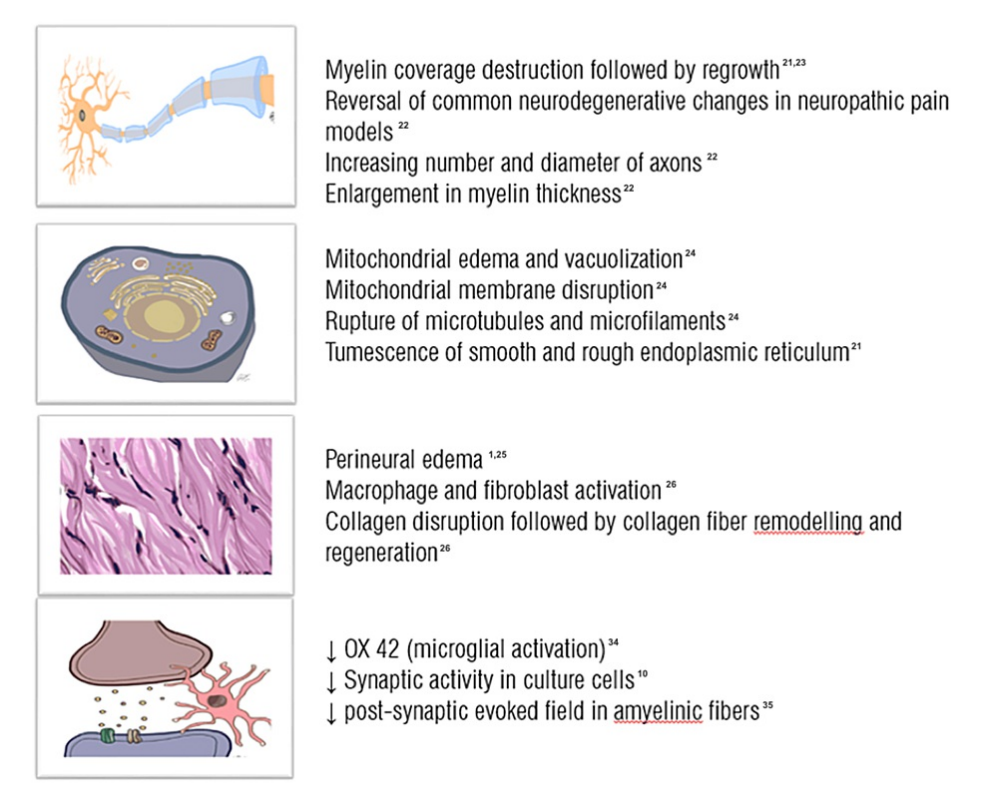
Microscopic changes after PRF in myelin sheath, organelles, connective tissue and microglial environment Source: Authors PRF: pulsed radiofrequency

PRF induces several microscopic changes. Cosman et al. used liver tissue and eggs to show that while conventional RF induced cell lesion, PRF was presumed to produce neuronal membrane changes [20]. A review of these microscopic changes can be seen in Figure 2.

Myelin Changes

Several studies showed the effects of PRF on myelin. Protasoni et al. in 2009 studied PRF effects in the acute phase (after one hour) in rat dorsal root ganglions and compared these findings with a control group [21]. They report that under light microscopy no major differences were found between the PRF-exposed group and controls, except for a slight interstitial edema in the treatment group. However, under transmission electron microscopy, they observed that T ganglia cells showed a smooth reticulum with enlarged cisternae and many vacuoles in the PRF group. Furthermore, there was abnormal and non-adherent myelin coverage with evidence of myelin envelope destruction in the nerve fibers in the PRF-treated rats (in 79.81% of fibers). Some myelinated fibers appeared delaminated or clumped in dark ovoid structures. The contralateral ganglion cells had only slight myelin envelope changes similar to the control group, probably due to technical artifacts (in 23.21% and 24.35% of fibers, respectively). These findings, according to the authors, could contribute to an improvement in pain transmission.

On the other hand, some studies showed that RF can decrease and reverse myelin degeneration, as seen in many neuropathic pain models. Jia et al., in 2016, analyzed 36 adult rats in a chronic constriction injury model (CCIM), in which the animals were randomized to receive either PRF or sham in the middle portion of the sciatic nerve ligation site 14 days after the initial injury [22]. The study revealed that the treatment group showed an improvement in both the number and diameter of axons as well as an increase in the myelin sheath thickness when compared to sham stimulation. Under electron microscopy, the sciatic nerve lesion site also showed an improvement in degeneration after PRF when compared to the control group.

In a study by Li et al. published in 2015, the authors studied the results of the application of PRF in the sciatic nerve of rats 14 days after being submitted to the CCIM [23]. The pain behavior was measured by the hindpaw mechanical withdrawal threshold (MWT) and thermal withdrawal latency (TWL). MWT is based on the application of monofilaments in animals with increasing force until a withdrawal response occurs, measuring the strength of von Frey's filament that causes a positive response. In TWL, the response to thermal stimuli is evaluated, and the response time to tolerance to thermal sensitivity is measured. Li et al. showed a gradual and significant improvement in the PRF-treated group when compared to the sham-treated group. Electron microscopy of the sciatic nerve of the sham-treated group revealed extensive demyelination and collagen fiber formation, while in the PRF-treated group, sparse demyelination with signs of fiber regrowth was observed. The authors concluded that PRF seems to improve the ultrastructural damage in some neuropathic pain models.

Tun et al. compared the results of ultrastructural changes in rat nerves submitted to conventional RF at 42^o^ C at different exposure times, conventional RF at 70^o^ C, and PRF [12]. They also found myelin sheath separation in a large number of myelinated axons after PRF exposure.

Organelle Changes

It is well known that axons have a large number of mitochondria to provide energy for neural transmission. Moreover, after the application of PRF in nerve fibers, many of these mitochondria show edema and vacuolization [21-23].

Erdine et al. studied rat sciatic nerve fibers that underwent PRF, comparing those results with the contralateral side which was punctured in a similar way but with no energy delivery [24]. The sciatic nerve fibers were analyzed 10 days after the application by transmission electron microscope. PRF was found to induce ultrastructural changes, characterized by mitochondrial swelling, mitochondrial membrane rupture, and vacuolization in some specimens. Additionally, microfilaments and microtubules showed a high level of disorganization and disruption. The authors found that C-fibers (unmyelinated) were more frequently damaged than A-delta or A-beta fibers.

Protasoni et al. also showed that in an acute phase (one hour after PRF application), the smooth and rough endoplasmic reticulum cisternae in rat ganglion cells were enlarged, with apparent signs of mitochondrial damage, characterized by its external membrane damage as well as diminished, enlarged or destroyed mitochondrial cristae [21]. A nuclear membrane or plasma membrane damage was seen in a few cases.

Changes in the Neural Connective Tissue

PRF seems to promote changes in the connective tissue, including epineural, perineural, and endoneural regions.

Vatanserver et al. studied sciatic samples from rats submitted to one of the following procedures: (a) Control (no procedure at all); (b) Sham (puncture with RF needle but no energy delivery); (c) Conventional RF at 40º C for 90 seconds; (d) Conventional RF at 80º C for 90 seconds; or (e) PRF at 45 Volts for 240 seconds, not exceeding 42º C [25]. The samples were obtained from the treatment region two days later and examined under both light and electron microscopes. The number of changes observed was greater in the conventional RF groups (highest in the 80º C group) than in the PRF group, but the latter also showed endoneural and perineural edema damage to the myelin fibers, as well as accumulation of neurofilaments and neurotubulous neural tissue changes represented by edema with increased interstitial spaces between interganglionar nervous fibers with normal periganglionic vessels [22]. The findings also show that glial cells may also be affected, as evidenced by the occurrence of macrophages containing necrotic tissue and cholesterol crystal surrounding myelin damage regions [23].

Choi et al., in 2013, showed that beyond slight damage and swelling of myelin, PRF also induced interstitial changes in the number and size of collagen fibers, mainly in the endoneurium and perineurium regions [26]. They found that in the sciatic nerve of rats, exposure to PRF induced a slight decrease in the amount of collagen fibers and a decreased immunoreactivity of collagen types I and III, with swelling of collagen fibril (increased diameters). After exposure to PRF, newly synthesized collagen formation appeared. Indeed, Podhajski et al. also reported PRF-induced subclinical findings in the rat dorsal ganglion root, including endoneural edema, fibroblast activation, and collagen deposition [1]. However, in this study, these changes were found to reverse after 21 days.

The effect of PRF has shown benefits inducing remodeling and proliferation of collagen fibers, improving wound healing in diabetic animal models [27], and probably in humans.

Effects in Animal Models of Allodynia

Several animal models of neuropathic pain induce allodynia, and PRF has been shown to improve the protection behavior. Li et al., for example, studied rats after chronic constriction injury and after exposure to PRF in the sciatic nerve [23]. Their results revealed that there was a significant improvement in allodynia as measured by the MWT and TWL. A beneficial effect of allodynia was also shown by Ösoylar et al. in neuropathic pain [17].

Tanaka et al. presented a comparative study on the effect of PRF in mechanical allodynia in rats induced with resiniferatoxin [28]. They analyzed both the ideal moment of the procedure and the optimal duration of exposure to RF, with results analyzed by MWT and TWL. They found that earlier exposure (one week after the pain induction) was related to a better outcome and that a longer time of PRF exposure was related to superior improvement (best results achieved significance with a minimum of six minutes of treatment).

Similarly, Aksu et al. showed that PRF applied on L5 and L6 dorsal roots promoted significant benefits concerning neuropathic pain in rabbits that had undergone sciatic nerve ligation, with effects lasting for at least four weeks [29]. Yang et al. proposed that excitatory amino acids may play a significant role in mechanical allodynia improvement [30].

Liu et al. showed significant improvement in thermo-hyperalgesia, mechano-allodynia, and mechano-hyperalgesia after PRF exposure in dorsal root ganglia in a model of neuropathic pain (chronic constriction injury) in rats [31]. Ren et al. showed a progressive improvement in rats treated with PRF in dorsal root ganglia when compared to sham [32]. A similar progressively positive outcome was also seen by Hailong et al. [5].

Yeh et al. showed that the application of both PRF at 45 Volts or at 60 Volts for six minutes had similar benefits with a prominent antiallodynic effect as well as significant inhibition of cold allodynia in neuropathic pain in rats [33]. These effects lasted for at least four weeks.

Cellular Effects

Park et al., in 2012, studied the effects of PRF in the dorsal root ganglia of rats submitted to L5 spinal nerve ligation with further distal transection [34]. A significant reduction of mechanical hypersensitivity was seen 12 days after the procedure in the treated group. Concomitantly, a reduction in immunoreactivity for OX42 was found, showing attenuation of microglial activation. However, cold hypersensitivity and glial fibrillary acidic protein (GFAP) were not affected by PRF (no major astrocytic change).

Cahana et al. studied synaptic transmission and cell survival in a model of hippocampal organotypic slice culture [10]. They studied how cells reacted in three situations: (a) PRF at a maximum of 38º C; (b) PRF at a maximum of 42º C; and (c) Continuous RF with a maximum temperature of 42º C. Inhibition of evoked synaptic activity in all groups was found with continuous RF showing more lasting effects. Furthermore, some degree of cell destruction at the region of the stimulating needle region was also found.

Huang et al. studied electrophysiological effects of PRF in both normal and chronic pain models in rats (spinal nerve ligation) [35]. They found that PRF selectively reduced the post-synaptic evoked field potential in C fibers, with minimal effects on A fibers.

Regulation of Peptides and Proteins

Cho et al. used a rat model of lumbar disc herniation at the L5 level and compared a PRF-treated group with a sham treatment group [36]. They found that in the first group, caudal PRF application attenuated calcitonin gene-related peptide (CGRP) immunoreactivity, not only at the L5 corresponding spinal dorsal horn but beyond this region (L6). The PRF group also showed decreased pain-related behavior that paralleled the reduced neuroglial expression. As CGRP is considered a marker of pain-related neurons, this study showed a positive effect of PRF in root compression and inflammation due to disc-induced nociception.

A similar finding was shown by Ren et al. who analyzed four groups of rats: groups A and B had their sciatic nerves isolated with no constriction, while groups C and D had their right sciatic nerves isolated and constricted (CCIM), which is known to induce CGRP overexpression [32]. Fourteen days after surgery, groups B and D were submitted to PRF exposure. The treated group D showed significant improvement in pain behavior, while a gradual decrease was found in CGRP mRNA expression to baseline levels (similar to groups A and B).

Hamann et al. described that in Wistar rats, the application of PRF either in the sciatic nerve at the mid-thigh or at the L4 anterior primary ramus just distal to the intervertebral foramen promoted a marked reduction in CGRP-positive neurons at the L4 dorsal root ganglia when compared to the control group [6]. However, this difference was not statistically significant. Furthermore, treated rats showed an increase in the upregulation of activating transcription factor 3 (ATF3) selectively in small- and medium-caliber neurons, evidencing nervous cell stress.

A study conducted by Vallejo et al. in 2013 analyzed the genetic expression induced by neuropathic pain as well as by further exposure to PRF [37]. The authors used the spared nerve injury model and compared spared nerve injury rats and controls 24 hours after PRF exposure or sham, respectively. Then, histological analysis was performed in three different sites in these rats: (a) the sciatic nerve, (b) the L5 dorsal root ganglia (L5-DRG), and (c) the spinal cord. The study showed that nerve injury induced upregulation of tumor necrosis factor-alpha (TNF-α) and interleukin-6 (IL-6) at the sciatic nerve. PRF induced many beneficial changes, including a downregulation of TNF-α and IL-6 to normal levels at the sciatic nerve and at L5-DRG and an upregulation of GABAB-R1, Na/K ATPase, and 5-HT3R at the L5-DRG as well as an upregulation of Na/K ATPase and c-Fos at the spinal cord.

The transcription and translation of neurotrophic factors have also been evaluated. Glial-cell-line-derived neurotrophic factor (GDNF) is an important neurotrophic factor found in the central and peripheral nervous systems [38]. In 2018, Hailong et al. found that in rat CCIMs, the delivery of PRF energy at the ligation site promoted an upregulation of the translation and transcription of GDNF, which in turn could have some positive effect on nerve cell repair and pain control [5].

Using the CCIM, Jia et al. found a significant improvement in pain behavior after PRF was applied in the chronic constriction injury site in rats. In addition, they showed an increased level of the expression of GDNF in the PRF-treated group [22].

Ion Channels

Liu et al.studied the effects of PRF on hyperpolarization-activated cyclic nucleotide-gated channels (HCNs) in a CCIM on Sprague-Dawley rats [31]. This group of voltage-gated channels showed increased expression with neuropathic pain, but their potential role has not been fully understood. Compared with controls, the authors found a higher expression of HCN1 and HCN2 in the dorsal root ganglia in the PRF group, concomitantly with an improvement in thermal hyperalgesia, mechano-allodynia, and mechano-hyperalgesia.

Inflammatory Proteins

One of the first described changes produced by PRF showing a biological neuromodulatory effect on the nervous system was the change in the proto-oncogene c-Fos. It is well known that c-Fos is a transcription factor related to many cellular changes, including cell differentiation, proliferation, and survival. In chronic pain, many studies have shown that after noxious stimulation, there is a massive increase in the expression of the protein Fos in the dorsal horn of the spinal cord, making it a possible neural marker of pain [39].

Researchers have also investigated immunoreactivity specifically. In 2002, Higuchi et al. analyzed changes after the application of different stimuli at the C6 dorsal root ganglia [40]. In their experiment, previously normal rats (no pain model in this sample) had a hemilaminectomy and were exposed to one of the following treatments: PRF, continuous RF up to 38° Celsius, or sham treatment. They found that only PRF was related to a significant increase in the immunoreactivity of c-Fos neurons in the ipsilateral dorsal horn lamina I and II, as seen three hours after this exposure, showing cellular activation, influencing the transcription of other genes and the response of a neuron to external stimuli. A few c-Fos-immunoreactive cells were also found at lamina V as well as in the contralateral dorsal horn I and II.

In 2005, Van Zundert et al. also studied c-Fos immunoreactivity after stimulation but over a long period [41]. They randomly exposed the cervical dorsal root ganglia of rats to one of the following treatments: (a) sham, (b) continuous RF at 67° Celsius, (c) PRF for two minutes, or (d) PRF for eight minutes. The dorsal horn was analyzed seven days after the intervention. Immunoreactivity to c-Fos was found to be significantly higher in the continuous RF and PRF groups than in the sham treatment group, and this effect was not dependent on temperature.

Further studies have shown that models of neuropathic pain, such as the constriction of neural roots, develop proapoptotic changes in astrocytes and microglia in superficial laminae. Extracellular signal-regulated kinase (ERK) and mitogen-activated protein kinase (MAPK) likely play an important role in these changes.

Lin et al. studied the activation of the MAPK family in a rat model of spinal nerve ligation before and after applying PRF in the L5 dorsal root ganglion [42]. They found that SNL induced signs of nociceptive behavior as well as increased p-ERK and p-p38 in the spinal dorsal horn one week later. After applying PRF, a significant reduction in nociceptive hypersensitivity was found concomitant with inhibition of ERK at neurons and the inhibition of p38 at microglia. The PRF group also demonstrated a significant suppression of TNF-α in the spinal dorsal horn. In 2018, Liu et al. published an experiment showing that PRF could decrease signs of pain behavior in rats undergoing the CCIM of neuropathic pain [43]. Further, PRF caused a decrease in the upregulation of interferon regulatory factor 8 (IRF8), microglial activation, and p38 phosphorylation in the spinal cord.

Yeh et al. found that in rats undergoing the spared nerve injury model, the group treated with PRF showed improvement in nociceptive behavior as well as an inhibition of the activation of ERK1/2 in the ipsilateral spinal dorsal horn analyzed on days 1 and 28 following application [33]. In addition, they showed that this effect was enhanced when a higher voltage was applied.

A further study, published by Lin et al., evaluated the exposure of different pulse waveforms and PRF parameters [44]. In a rat model of ligation of the L5 spinal nerve (Chung model), the authors evaluated pain behavior and c-Fos and pp38 levels in the spinal cord. They found that the sinusoidal group had a higher response than the square wave. In 2019, Xu et al. showed that in rats undergoing a spared nerve injury model of neuropathic pain, PRF could partially reverse mechanical and thermal hypersensitivity and significantly decrease microglial activation, brain-derived neurotrophic factor (BDNF) upregulation, and phosphatidylinositol 3-kinase (PI3K) and p38 phosphorylation [45].

The experiments from Huang et al. using PRF either in normal rats or those with a previous spinal nerve injury showed a long-lasting inhibition of injury-induced ERK-MAPK activation in spinal neurons and astrocytes [35].

Ramzy et al. observed that after PRF, there was an improvement in the hind limb posture that was changed after the constriction of the sciatic nerve [46]. They also showed an association with the decrease in IL-6 and TNF observed most significantly when a longer treatment with PRF (for eight minutes) was used. An inhibition of TNF-α in the neuroglia was also seen after PRF exposure

Choi et al. showed, in rat models, a positive regulation of proteins and inflammatory cytokines, such as GFAP, TNF- α, IL-6, COX-2, iNOS, NF-kB, MCP-1 and MIP 1α proteins after RF, when an upregulation was seen seven days after the PRF procedure [47]. However, on day 30, the levels of these proteins and cytokines were normalized, although the behavioral response remained improved.

The effect of RF on synaptic transmission in the spinal cord was described by Liu et al., who showed that the application of Freund complete adjuvant caused a reduction in the expression of the *KCC2* gene related to gamma-aminobutyric acid (GABA) inhibitory control, an important determinant for the efficacy of inhibitory neurotransmission in the spinal cord. In addition, post-RF KCC2 levels were elevated with a partial restoration of GABA synaptic activity [48].

Das et al. carried out an interesting study on the role of modulation of PRF on the inflammatory response in 10 humans with root pain without previous spinal surgery [49]. In their sample, cytokines and cells in the cerebrospinal fluid after 10 weeks of PRF treatment were analyzed and depicted a decreased cell frequency in CD56+, CD3, and NK cells. In addition, there was a reduction in IFN-γ levels and an increase in CD8+ and IL-6 cell frequency. The authors also showed a positive correlation between IL-17 concentrations and postoperative pain.

Changes in Neuromodulatory Pathways

Wu et al. analyzed the results of PRF in rats subjected to sciatic nerve ligation [50]. They found that the levels of met-enkephalin (measured by immunoassay) were increased up to two days after the procedure throughout the spinal cord, as opposed to what was found in the non-RF group. Such elevation may suggest endogenous RF-stimulated control. Laboureyras et al. found that in rats with neuropathic pain, after PRF, there was a magnification of the analgesic effect following the administration of morphine opioid treatment [51].

Hagiwara et al. observed that after PRF treatment in rats, the effects may partially be inhibited by the intrathecal administration of serotonergic and noradrenergic antagonists, suggesting the possibility that some degree of the analgesic effects of PRF would be due to the inhibitory control of the descending pathways [52].

Miao Fu et al. used a CCIM of neuropathic pain in rats and compared a sham-treated group with a PRF-treated group 28 days later [53]. They showed that PRF induced a decrease in the protein expression of the purinergic receptor ligand-gated ion channel 3 (P2X3) when compared to the sham treatment, either in the DRG or in the spinal dorsal horn (27.8% lower in the DRG and 35.6% lower in the spinal dorsal horn), suggesting that this mechanism may explain part of the analgesic effect of PRF.

Neurotransmitters

An additional study published by Huang et al. induced diabetic neuropathy after intraperitoneal injection of streptozotocin [54]. Some days later, the rats were submitted to pulsed radiofrequency at the L5 and L6 roots. They found significant improvement in responses to thermal, mechanical and cold stimuli. Moreover, they found a decrease in glutamate levels in the cerebrospinal fluid by microdialysis techniques.

Yang et al. also performed a study analyzing the levels of excitatory neurotransmitters [30]. They found a decrease in aspartate, glutamate, and citrulline levels in the dorsal root ganglion in rats subjected to pulsed radiofrequency.

A review of the effects of PRF on molecular levels, ion channels, and receptors can be found in Table 1.

**Table 1:** Summary of effects of pulsed radiofrequency at molecular, ion channels, and receptors. CGRP: calcitonin gene-related peptide; ATF-3: activating transcription factor 3; NFkB: nuclear factor-kappa B; IRF-8: interferon regulatory factor 8; ERK; extracelluar-signal-regulated protein kinase; p38 MAPK: p38 mitogen-activated protein kinase; TNF-α: tumor necrosis factor-alpha; IL-6: interleukin-6; GDNF glial cell-derived neurotrophic factor; c-Fos: Fos proto-oncogene; GFAP: glial fibrillary acidic protein; NA,K-ATPase: sodium-potassium pump; KCC2: potassium chloride co-transporter 2; COX-2: cyclooxygenase-2; iNOS: inducible nitric oxide synthase; HCN1 and HCN2: hyperpolarization-activated cyclic nucleotide-gated potassium channel 1 and 2, respectively; GABA B-R1: gamma-aminobutyric acid type B receptor subunit 1 Superscripts are the references

TARGET	EFFECTS
Neuropeptides	> CGRP in dorsal horn^ [32, 36]^
Transcription factors	> ATF-3 | Normalization of NFkB | < IRF-8^ [6, 42]^
Intracellular signaling	< ERK and p38 MAPK phosphorylation | Inhibition of ERK 1/2 ^[32, 35, 42-44]^
Cytokines	Normalization of TNF-α and IL-6 levels ^[37, 46, 49]^
Neurotrophic factors	> GDNF^ [5]^
Proto-oncogenes	> c-Fos ^[39-41, 44]^
Proteins	Positive regulation of GFAP | > NA,K-ATPase | > KCC2 with normalization of GABA synaptic activity ^[47]^
Enzymes	Normalization of COX-2 and iNOS ^[47]^
Ion channels/Receptors	> HCN1 and HCN2 | > GABA B-R1 ^[31, 37]^

Other Effects

Some studies have shown that PRF may induce tissue regeneration at different sites. In the near future, it is likely that many other beneficial effects will be described.

Herman et al. conducted a study in animals with fecal incontinence after the destruction of the pudendal nerve [55]. The authors studied 16 pigs in three groups (1. Fecal incontinence + RF; 2. Fecal incontinence with no RF; and 3. Control group with no incontinence and no treatment). The study showed that nonablative RF produced beneficial hyperplasia in the anal sphincter without local scar formation.

Yang et al. studied rats with neuropathic pain due to L5 spinal nerve ligation and compared them with rats undergoing spinal nerve ligation plus PRF [56]. They found that mechanical hyperalgesia was found on all analyzed days (1, 3, 6, 14, and 28 days) in the spinal nerve ligation group, and PRF decreased this hypersensitivity. Furthermore, PRF could reverse the effect of the autophagy-related protein LC3 and autophagic-related receptor P62. They propose that regulating the level of autophagy in the spinal dorsal horn is an important mechanism related to PRF.

Another PRF application was reported by Lin et al. [57]. PRF was applied in the T2 ganglion, showing a modulation of the effect of palmar hyperhidrosis, including a 63.6% reduction in sweating. Chang et al. reported two patients with spinal cord injury with spasticity whose spasticity improved after PRF in the dorsal root ganglion of L2, L3, and S1 for six months [58]. The authors suggest that PRF could enhance the inhibitory control of the spinal reflex and decrease the excitatory afferent input entering the spinal cord, reducing the flow of nociceptive information. Further research involving a greater number of cases is needed to confirm these findings.

In addition, RF is not limited to the central nervous system, as described by Li et al. [27]. In their work in diabetic rats, the healing process of wounds was evaluated and observed through histopathological studies, and they concluded that RF accelerated the granulation process of the skin and stimulated the organization of collagen [27]. In the sperm of adult rats under experimental conditions of PRF with 220 MHz, a disruption in the secretory function of Leydig cells and in apoptosis was found, and testis field exposure could reduce sperm quality in rats [59].

Erdine et al. stated that PRF is non-destructive with neuromodulatory effects based on the following findings: (a) PRF is effective at temperatures below the heat-lesion threshold; (b) there is no significant sensory loss; and (c) a typical PRF procedure does not promote pain, even when touching sensory structures [24].

Finally, Van Boxem et al. reviewed the literature up to 2014, explaining the mechanisms of PRF through cellular and molecular mechanisms [60]. It was the only revision paper elucidating the mechanisms of RF for pain treatment in the literature until now. Since that time, many other effects have been added.

## Conclusions

Despite numerous studies showing the effectiveness of PRF in the treatment of multiple diseases with chronic pain, the exact mechanism of action of the neural changes remains a matter of debate. Nonetheless, based on the many studies shown here, we can agree that PRF may be considered as a valid neuromodulatory procedure. PRF has been shown to be nondestructive based on the following findings that have thus far emerged from the relevant research: (a) PRF is effective in temperatures below the heat-lesion threshold; (b) there is no significant sensory loss; and (c) a typical PRF procedure does not promote pain, even when touching sensory structures. PRF induces multiple changes that can be related to its analgesic effect.
